# Artificial intelligence for the prediction of acute kidney injury during the perioperative period: systematic review and Meta-analysis of diagnostic test accuracy

**DOI:** 10.1186/s12882-022-03025-w

**Published:** 2022-12-19

**Authors:** Hanfei Zhang, Amanda Y. Wang, Shukun Wu, Johnathan Ngo, Yunlin Feng, Xin He, Yingfeng Zhang, Xingwei Wu, Daqing Hong

**Affiliations:** 1 School of Medicine, University of Electronic Science and Technology of China, Chengdu, China; 2 Department of Nephrology, Sichuan Provincial People’s Hospital, University of Electronic Science and Technology of China, Chengdu, China; 3 The faculty of medicine and health sciences, Macquarie University, Sydney, NSW Australia; 4 Concord Clinical School, University of Sydney, Sydney, Australia; 5 Department of Nephrology, Affiliated Hospital of Southwest Medical University, Luzhou, China; 6 Department of Pharmacy, Sichuan Provincial Peoples Hospital, School of Medicine, University of Electronic Science and Technology of China, Chengdu, China; 7 Renal Department and Nephrology Institute, Sichuan Provincial People’s Hospital, School of Medicine, University of Electronic Science and Technology of China, Chengdu, China

**Keywords:** Artificial intelligence, Machine learning, Acute kidney injury, Acute kidney failure, Perioperative period

## Abstract

**Background:**

Acute kidney injury (AKI) is independently associated with morbidity and mortality in a wide range of surgical settings. Nowadays, with the increasing use of electronic health records (EHR), advances in patient information retrieval, and cost reduction in clinical informatics, artificial intelligence is increasingly being used to improve early recognition and management for perioperative AKI. However, there is no quantitative synthesis of the performance of these methods. We conducted this systematic review and meta-analysis to estimate the sensitivity and specificity of artificial intelligence for the prediction of acute kidney injury during the perioperative period.

**Methods:**

Pubmed, Embase, and Cochrane Library were searched to 2nd October 2021. Studies presenting diagnostic performance of artificial intelligence in the early detection of perioperative acute kidney injury were included. True positives, false positives, true negatives and false negatives were pooled to collate specificity and sensitivity with 95% CIs and results were portrayed in forest plots. The risk of bias of eligible studies was assessed using the PROBAST tool.

**Results:**

Nineteen studies involving 304,076 patients were included. Quantitative random-effects meta-analysis using the Rutter and Gatsonis hierarchical summary receiver operating characteristics (HSROC) model revealed pooled sensitivity, specificity, and diagnostic odds ratio of 0.77 (95% CI: 0.73 to 0.81),0.75 (95% CI: 0.71 to 0.80), and 10.7 (95% CI 8.5 to 13.5), respectively. Threshold effect was found to be the only source of heterogeneity, and there was no evidence of publication bias.

**Conclusions:**

Our review demonstrates the promising performance of artificial intelligence for early prediction of perioperative AKI. The limitations of lacking external validation performance and being conducted only at a single center should be overcome.

**Trial registration:**

This study was not registered with PROSPERO.

**Supplementary Information:**

The online version contains supplementary material available at 10.1186/s12882-022-03025-w.

## Introduction

Acute Kidney Injury (AKI) is a clinical syndrome characterised by a sudden decrease in glomerular filtration rate, defined by a rapid increase in serum creatinine, decrease in urine output, or both [1]. Noteworthy, AKI in the perioperative period is one of the most serious yet under-recognised complications, associated with increased risk of morbidity and mortality, chronic kidney disease, long-term adverse events, and increased cost and resource utilisation [2–4]. Nephrologists should recognise the huge medical burden.

Despite remarkable improvements in the identification of high-risk patients [5], assessment of AKI is still based on two relatively non-specific markers that may lack utility in discriminating patients with incipient AKI: serum creatinine (SCr) and urine output (UO) [6]. Urine output is a sensitive detection tool for identifying acute kidney injury, but probably confounded by multiple factors [7]. One randomized prospective study examined the relationship between fluid administration and intraoperative urine output and its correlation with postoperative acute kidney injury. The authors failed to find a correlation between intraoperative low urine output and postoperative acute kidney injury in 102 bariatric surgery patients receiving high- or low-volume of lactated Ringer’s solution [8]. Moreover, SCr detected may vary in critically ill patients (e.g., severe hepatic disease) or by diet (e.g., food rich in proteins). In addition, sarcopenia and sepsis lead to reduced creatine release and decreased creatinine production [6]. This suggested that there remained many difficulties in diagnosing perioperative AKI and it was of high importance to develop a more accurate and timely diagnostic approach [6].

Artificial intelligence (AI) is a fast-growing field, and its applications to acute kidney injury can reform the approach to diagnosing and managing this clinical syndrome. There are numerous AI algorithms (random forest, Bayesian network, Gradient boosting machines, etc.) to choose from to support predictive models which can automatically trigger an electronic alert to physicians [9]. In previous studies, AI models demonstrate improved accuracy in identifying patients at risk of developing AKI, as well as early recognition of subclinical AKI, compared with traditional multivariate regression models [10]. However, there is no quantitative synthesis of the diagnostic accuracy of these methods. Researchers have tried different ways, including but not limited to expanding sample sizes, use of real-time predictive analytics, finding novel biomarkers, and optimising algorithms, in an attempt to raise diagnostic accuracy but have received conflicting results [11, 12].

We conducted a systematic review and meta-analysis to quantitatively analyse the diagnostic accuracy of the AIs in detecting acute Kidney Injury during the perioperative period and investigated the factors that affected diagnostic accuracy.

## Methods

### Data sources and searches

Two independent evaluators searched PubMed, Embase, and the Cochrane Library using combined free texts and MeSH terms relating to the perioperative period, acute kidney injury, and AI (prior to October 2021). The abstracts of all identified studies were reviewed to exclude irrelevant articles. Full-text reviews were conducted to determine whether the inclusion criteria were satisfied in all the studies. We also manually checked the reference lists of relevant publications including reviews and commentaries to include eligible studies. Disagreements were resolved by a discussion between two evaluators. Additional file 1 shows the detailed search strategy.

### Selection criteria

Studies were eligible if they met the following inclusion criteria: (1) AKI was defined using consensus criteria such as RIFLE, AKIN, and KDIGO, or studies with clear AKI definitions; (2) the main outcome was the onset of AKI during the immediate pre-operative period until the time of discharge; (3) application of the AI algorithm for the prediction of perioperative acute kidney injury; (4) inclusion of diagnostic performance indices of the AI algorithm, including specificity, sensitivity, positive likelihood ratio (PLR), negative likelihood ratio (NLR), positive predictive value (PPV), negative predictive value (NPV), or the figure of the area under the receiver operating characteristic curve, which enables the construction of a 2 × 2 diagnostic table; and (5) human adult subjects.

The exclusion criteria were the studies that were not original studies such as letters, comments, editorials, protocols or reviews.

### Data extraction and quality assessment

The data that was extracted independently by two investigators included study characteristics (authors and year of publication); characteristics of the sample set (sample size, age, sex, and type of surgery); characteristics of the index test (external validation, number of predictors, and type of AIs); characteristics of reference standard; and accuracy data (number of true positives, true negatives, false positives, and false negatives). If different types of models were compared in the same study, we only included the model which had the highest diagnostic accuracy. When original studies reported the sensitivity and specificity under multiple thresholds, we extracted the accuracy data under the threshold with the largest Youden’s index, defined as the sum of sensitivity and specificity minus one. If both the internal validation and external validation were performed, the two-by-two data of the latter was extracted, because of better generalisability.

We assessed the methodological quality in 20 signalling questions in 4 key domains: participants, predictors, outcome, and analysis of each study using the Prediction model Risk Of Bias Assessment Tool (PROBAST), which is a risk of bias assessment tool designed for systematic reviews of diagnostic or prognostic prediction models [13, 14]. According to the signal problem and the author’s judgment, each of the domains was divided into “high”, “low” and “unclear”. Overall risk of bias is graded as low risk when all domains are considered low risk, and overall risk of bias is considered high risk when at least one of the domains is considered high risk.

### Data synthesis and analysis

Extracted two-by-two data were first graphically shown in the forest plot with the point estimate of sensitivity and specificity and their 95% confidence intervals (Cis). To remove the effect of a possible heterogeneous threshold, we conducted a quantitative random-effects meta-analysis using Rutter and Gatsonis hierarchical summary receiver operating characteristics (HSROC) model to combine summary receiver operating characteristic curves (SROC) curve which was the standard method for meta-analysing diagnostic studies reporting pairs of sensitivity and specificity [15]. This method comprehensively considers the effect of diagnostic tests under different diagnostic thresholds and converts the diagnostic odds ratio (DOR) by the sensitivity and specificity of each pair as the only metric of diagnostic analysis [16].

Subgroup analysis and meta-regression were used to explore the potential heterogeneity. The following pre-specified subgroup analyses were performed based on AI algorithms, surgery type, number of patients, external validation, diagnostic criteria, and methodological quality of included studies. We regarded the factor as a source of heterogeneity if the coefficient of the covariate was statistically significant (*P* < 0.05). Because the Metandi and Midas package of STATA required a minimum of four studies to conduct the diagnostic test accuracy meta-analysis (reference), if less than four studies were enrolled in the subgroup analysis, Meta-DiSc 1.4 using the ‘Moses-Shapiro-Littenberg method’ was used (reference).

We performed sensitivity analysis to evaluate the robustness of our main outcomes by exploring the effect of excluding one study at a time and used Deek’s funnel plot [17] to assess the presence of publication bias. All the data analysis were conducted in STATA (version 16.0) with the two-tailed probability of type I error of 0.05 (*α* = 0.05).

## Results

### Identification of relevant studies

**Fig. 1:**
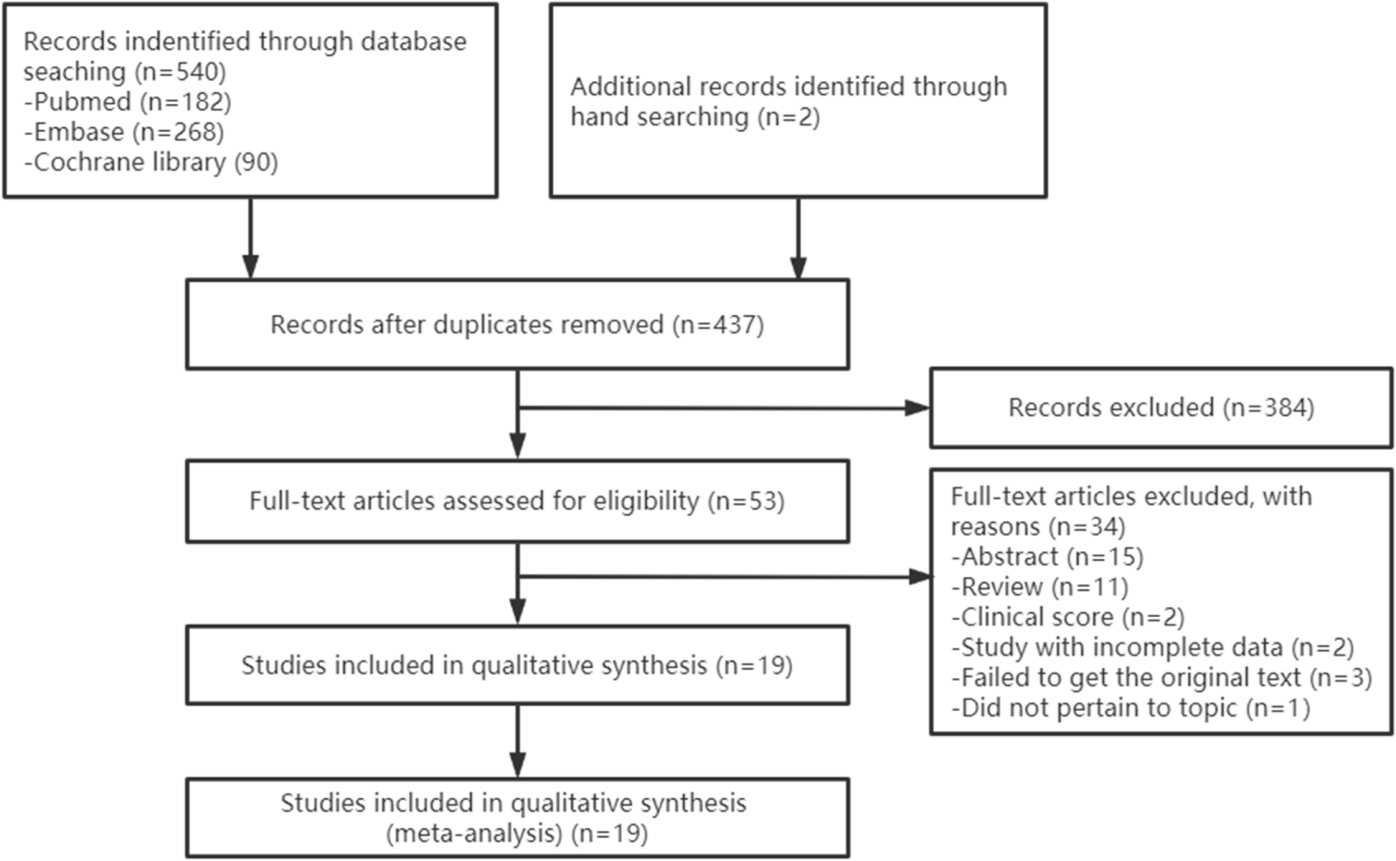
Flow diagram of the identification of relevant studies

A total of 540 articles were identified by searching three electronic databases. Among them, 105 were duplicate studies, and 384 were excluded during the initial screening by reviewing titles and abstracts. The full texts of the remaining 53 articles were thoroughly reviewed. Among these, 34 studies were excluded from the final analysis due to the following reasons: abstract (*n* = 15), review (*n* = 11), clinical score (*n* = 2), study with incomplete data (*n* = 2), failed to get the original text (*n* = 3) and did not pertain to topic (*n* = 1; the topic of this article was automated identification of the electronic medical record). The remaining 19 studies were included in the final analysis, which was shown in Fig. 1.

### Characteristics of eligible studies

The total number of subjects tested in the included studies was 304,076, with the sample size ranged from 109 to 96,653 [18–36].

Seventeen studies described the demographic characteristics of their study population, of whom the mean age was 37 to 71 years old and the percentage of males was 16 to 88% [18, 20, 21, 23–30, 36].

The included studies were categorized based on the type of the surgery participants received, including cardiothoracic surgery, any inpatient operative procedure, liver transplantation, total knee arthroplasty [18–36].

Enrolled studies presented the performance of the AI algorithms with test dataset (internal validation), and there were only four studies [22, 27, 28, 35] that presented the performance of external validation. Nine studies [22–26, 29, 33–35] established the AI algorithm based on the gradient boosting machine (GBM), three studies [18, 20, 36] established random forest (RF)-based algorithms, three studies [21, 28, 30] established two types of artificial neural network (ANN)-based algorithms, one study [27] established Bayesian network (BN)-based algorithm, one study [32] established decision-tree (DT)-based algorithm, one study [31] established an ensemble algorithm, and another study even conducted a novel machine learning risk algorithm [19] called: MySurgeryRisk .

Fifteen studies applied the Kidney Disease Improving Global Outcomes (KDIGO) definition for AKI [18–20, 22, 23, 25–28, 30, 31, 36]. Among these, some used serum creatinine changes only to define AKI while urine output criteria were not adopted [22, 24, 26, 30, 35]. Two studies applied the Acute Kidney Injury Network (AKIN) criteria [21, 24].

These characteristics (modifiers) were evaluated as potential sources of heterogeneity through subgroup analysis and meta-regression. (Table 1) shows the detailed characteristics of the studies.

**Table 1:** Clinical characteristics of the included studies

Author, year	Number of patients	External validation	Type of surgery	CCI, mean ± SD/median (range)	ASA class 3 + 4 (%)	CKD (%)	AKI definition	Age (y), mean ± SD/median (range)	Male (%)	Model type	Predictors
AdhikariI,2019 [18]	2911	No	Any type of inpatient operative procedure	2 (1, 3)	NR	12	KDIGO	60 (49, 69)	60	RF	69
Bihorac,2019 [19]	51,457	No	Any type of inpatient operative procedure	NR	NR	NR	KDIGO	NR	NR	MySurgeryRisk	16
Filiberto,2021 [20]	1631	No	Cardiovascular surgery	5 (3, 7)	NR	30	KDIGO	68 (59, 75)	66	RF	367
Ko,2020 [22]	455	Yes	Total joint arthroplasty	NR	8	NR	KDIGO	71 ± 6	16	GBM	6
Lee(1),2018 [23]	363	No	Liver transplantation	NR	NR	NR	AKIN	53 (48,60)	68	GBM	20
Lee(2),2018 [24]	1005	No	Cardiovascular surgery	NR	NR	6	KDIGO	64 (55,71)	73	GBM	72
Lei,2019 [25]	8494	No	Noncardiac surgery	NR	53	NR	KDIGO stage I	58 ± 16	54	GBM	339
Lei,2020 [26]	270	No	Cardiovascular surgery	NR	NR	NR	KDIGO	48 ± 10	74	GBM	20
Li,2020 [27]	1894	Yes	Cardiovascular surgery	NR	NR	NR	KDIGO	56 ± 13	58	BN	12
Meyer,2018 [28]	5898	Yes	Cardiovascular surgery	NR	NR	NR	KDIGO stage III	68 (59, 76)	69	ANN	52
Penny-Dimri,2021 [29]	96,653	No	Cardiovascular surgery	NR	NR	NR	Self-defined+RRT	NR	73	GBM	56
Rank,2020 [30]	350	No	Cardiovascular surgery	NR	NR	NR	KDIGO stage I or II	69 ± 14	67	ANN	96
Tseng,2020 [31]	202	No	Cardiovascular surgery	NR	80	NR	KDIGO	63 (53,71)	65	Ensemble	94
Xin,2021 [32]	109	No	Liver transplantation	NR	NR	NR	KDIGO	54 ± 9	83	DT	NR
Xue,2021 [33]	106,870	No	Any type of inpatient operative procedure	NR	54	5	KDIGO	NR	NR	GBM	711
Yayac,2021 [34]	20,800	No	Total joint arthroplasty	0.4 (0.9)	NR	2	KDIGO	66 ± 11	55	GBM	41
Zhang,2021 [35]	195	Yes	Liver transplantation	NR	NR	NR	KDIGO	47 ± 10	88	GBM	111
Zhou,2020 [36]	212	No	Cardiovascular surgery	NR	NR	NR	RRT	37 ± 10	70	RF	7

*CCI* Charlson’s comorbidity index, *CKD* Chronic kidney disease, *ANN* artificial neural network, *GBM* gradient boosting machine, *RF* random forest, *BN* Bayesian network, *DT* decision tree, *RRT* renal replacement therapy, *TP* true positive, *FP* false positive, *FN* false negative, *TN* true negative, *NR* not reported

### Methodological quality of the studies (Fig. 2)

Among the 19 studies [18–36] in the final analysis, 4 studies [19, 26, 33, 34] showed low risk of bias, 2 studies [27, 30]showed unclear risk of bias, and 13 studies [18, 20–25, 27–29, 31, 32, 36] showed high risk of bias.

**Fig. 2:**
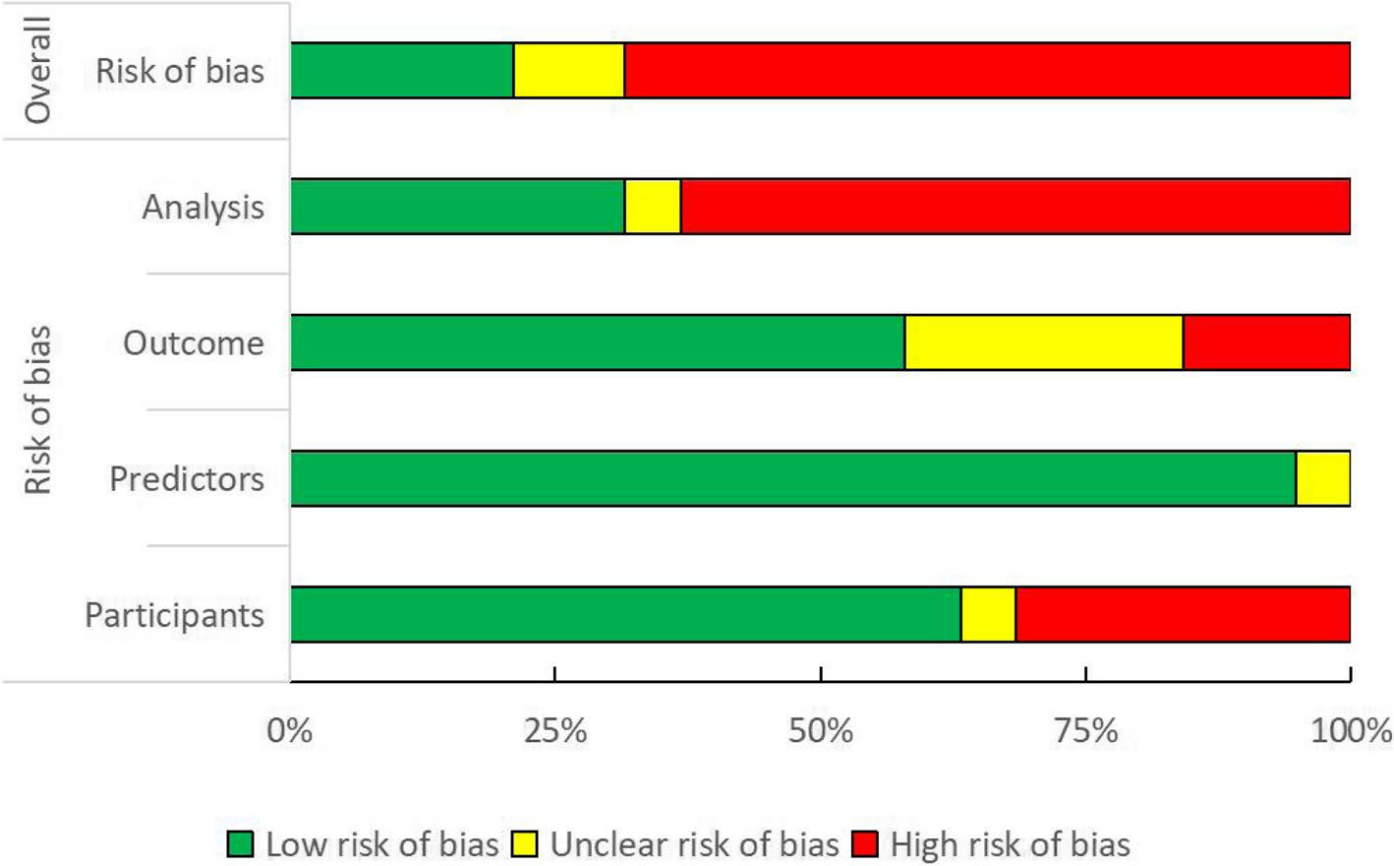
Risk of bias assessment (using PROBAST) based on four domains

Regarding the participants domain, the risk of bias was high in 6 studies [18, 21, 22, 25, 27, 34] because their participant data were from existing sources, such as existing cohort studies or routine care registries and didn’t appropriately adjust baseline hazards or registry outcome frequency in the analysis. The risk of bias was unclear in one due to insufficient information describing the sampling method in external validation [27]. Models developed using data without restricted inclusion criteria tend to show lower discriminative ability.

Concerning the predictors domain, we considered the risk of bias unclear in one study [32] because the details of the predictors were not reported.

In terms of the outcomes, 15 studies [18–20, 22, 23, 25–28, 30, 31, 36] applied the Kidney Disease Improving Global Outcomes (KDIGO) definition for AKI, but we considered the risk of bias unclear in five studies [22, 23, 25, 30, 35] because they utilised creatinine changes only. The risk of bias was high in one study [28] because only patients with severe AKI were enrolled. In addition, two studies [29, 36] which used their own criteria for AKI were also considered to have high risk of bias. These differences in outcome determination affect the estimated associations between predictors and outcome and thus the predictive accuracy of the diagnostic models [14].

The most concerning issue regarding “analysis” was the high risk of bias in majority of the included studies. The risk of bias in 12 studies [18, 20–24, 28, 29, 31, 32, 35, 36] was considered high and primarily related to unreasonable number of participants (e.g., EPV < 10 or small sample sizes), follow-up losses, and the absence of calibration and discrimination.

Overall, studies [18, 20–25, 27–29, 31, 32, 36] with high risk in at least one of the four domains were rated as low methodological quality in the diagnostic test accuracy of artificial intelligence for the prediction of acute kidney injury during the perioperative period (Fig. 2, Additional file 2).

### Diagnostic test accuracy of artificial intelligence for the prediction of acute kidney injury during perioperative period

**Fig. 3:**
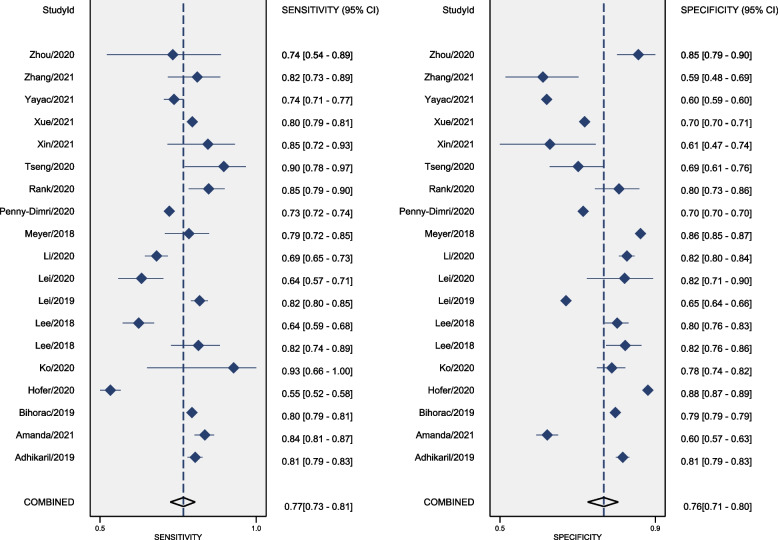
Forest plots of sensitivity and specificity of artificial intelligence algorithm for the prediction of Acute Kidney Injury during the perioperative period

**Fig. 4:**
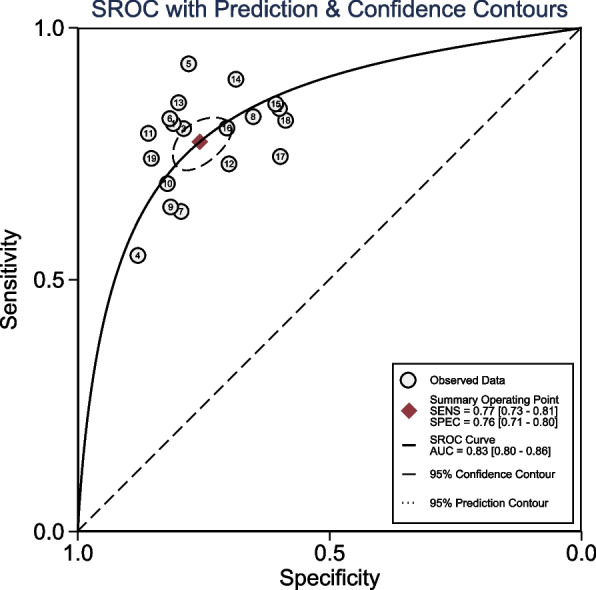
Summary receiver operating characteristic curve with 95% confidence region for the prediction of AKI during the perioperative period

**Fig. 5:**
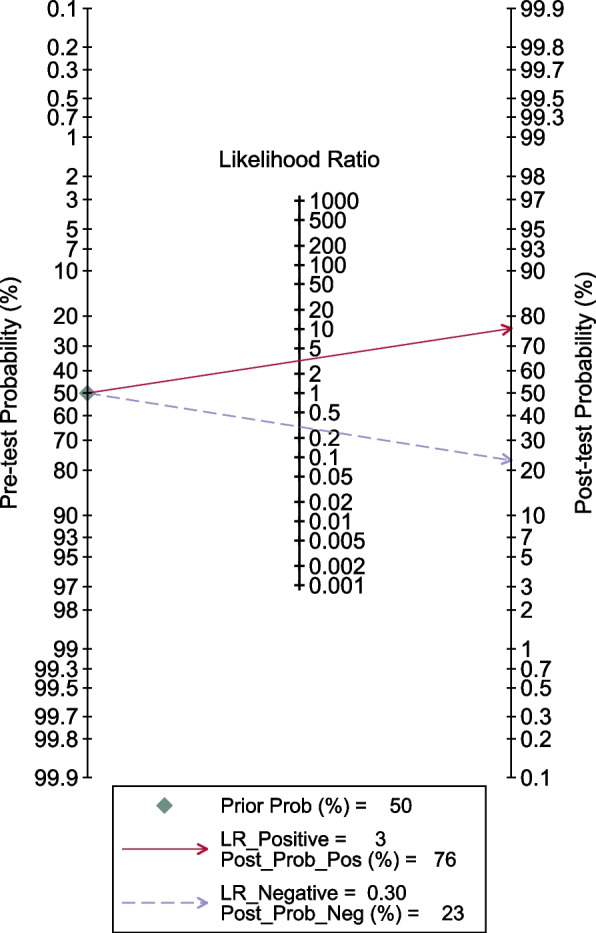
Fagan normogram for the prediction of AKI during the perioperative period

The Fig. 3 showed the paired forest plot for sensitivity and specificity with the corresponding 95% CIs for each study. The SROC curve, with a 95% confidence region, was illustrated in Fig. 4. The following summarised estimates using the HSROC model were also calculated: sensitivity 0.77 (95% CI: 0.73 to 0.81), specificity 0.75 (95% CI: 0.71 to 0.80), positive likelihood ratio 3.2 (95% CI: 2.7 to 3.7), negative likelihood ratio 0.30 (95% CI: 0.26 to 0.35), and diagnostic odds ratio 10.7 (95% CI 8.5 to 13.5). To investigate the clinical utility of AI, a Fagan nomogram was generated. Assuming a 50% prevalence of AKI during the perioperative period, the Fagan nomogram shows that the posterior probability of AKI was 76% if the test was positive, and the posterior probability of the absence of AKI was 23% if the test was negative (Fig. 5).

### Exploring heterogeneity with Meta-regression and subgroup analysis

The shape of the SROC curve was symmetric (Fig. 4). However, we observed a medium positive correlation after logit transformed TPR and FPR (Spearman correlation coefficient = 0.48), and an asymmetric parameter, β, with a significant *P*-value (*P* = 0.036) indicating threshold heterogeneity among the studies.

**Fig. 6:**
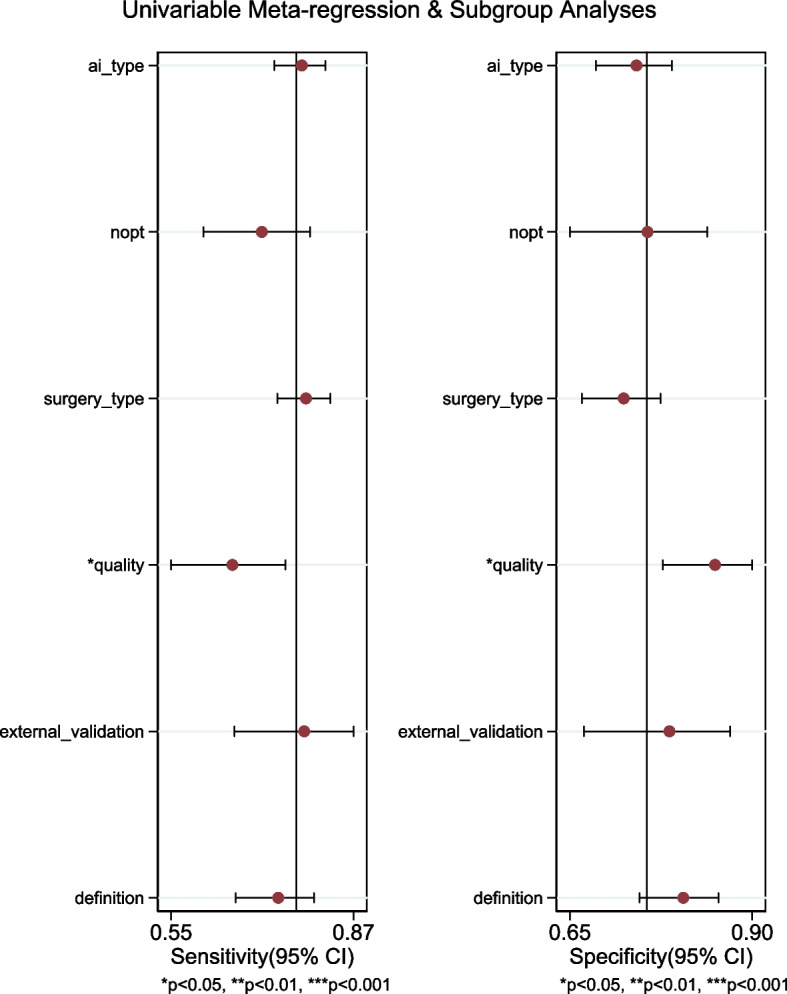
Meta-regression for the reason of heterogeneity in the diagnostic test accuracy meta-analysis. Nopt:number of patients

The heterogeneity was not found among the included studies in the joint model of meta-regression (AI algorithms [*P* = 0.58], number of included patients [*P* = 0.22], type of surgery [*P* = 0.17], methodological quality [*P* = 0.93], external validation [*P* = 0.69], the definition of AKI [*p* = .14] Fig. 6).

(Table 2) shows the detailed results of subgroup analysis exploring the potential source of between-study heterogeneity.

**Table 2:** Summary of diagnostic test accuracy and subgroup analysis of the included studies

Subgroup	Number of included studies	Sensitivity (95% CI)	Specificity (95% CI)	PLR	NRL	DOR
Type of AI algorithms						
GBM	9	0.77 (0.76–0.78)	0.69 (0.69–0.69)	2.7 (2.4–3.0)	0.34 (0.29–0.41)	7.8 (6.1–10)
RF	3	0.82 (0.80–0.84)	0.74 (0.72–0.76)	3.5 (1.9–6.4)	0.25 (0.22–0.27)	13 (6.5–26)
ANN	3	0.62 (0.59–0.64)	0.87 (0.86–0.87)	4.9 (4.0–6.0)	0.29 (0.14–0.60)	16 (7.8–34)
Number of patients						
< 1000	8	0.79 (0.76–0.82)	0.77 (0.75–0.79)	3.4 (2.6–4.3)	0.25 (0.17–0.36)	14 (9.0–21)
≥ 1000	11	0.78 (0.78–0.79)	0.71 (0.71–0.71)	3.1 (2.7–3.7)	0.33 (0.28–0.39)	9.6 (7.3–13)
Type of surgery						
Cardiovascular surgery	9	0.73 (0.72–0.74)	0.71 (0.71–0.71)	3.4 (2.7–4.4)	0.33 (0.28–0.38)	11 (8.0–15)
Any type of inpatient operative procedure	4	0.79 (0.78–0.80)	0.73 (0.73–0.73)	3.7 (2.8–5.0)	0.31 (0.23–0.41)	12 (9.0–17)
Liver transplantation	3	0.82 (0.77–0.87)	0.73 (0.69–0.78)	2.7 (1.6–4.6)	0.26 (0.20–0.34)	11 (4.9–23)
Total joint arthroplasty	2	0.75 (0.72–0.78)	0.60 (0.60–0.61)	2.8 (1.2–6.3)	0.27 (0.07–1.01)	11 (1.2–110)
Methodological quality						
Low quality	13	0.73 (0.72–0.74)	0.72 (0.72–0.72)	3.4 (2.7–4.2)	0.32 (0.26–0.38)	11 (8.2–15)
Unclear quality	2	0.72 (0.70–0.75)	0.82 (0.80–0.84)	3.9 (3.5–4.4)	0.27 (0.14–0.54)	15 (6.8–32)
High quality	4	0.80 (0.80–0.80)	0.71 (0.71–0.71)	2.6 (2.0–3.5)	0.30 (0.26–0.35)	8.6 (5.6–13)
External validation						
No	15	0.78 (0.78–0.79)	0.71 (0.71–0.71)	3.1 (2.7–3.6)	0.31 (0.26–0.36)	10 (8.0–13)
Yes	4	0.72 (0.69–0.75)	0.85 (0.84–0.85)	3.7 (2.5–5.6)	0.30 (0.22–0.42)	13 (7.0–24)
AKI definition						
KDIGO	14	0.80 (0.79–0.80)	0.71 (0.71–0.71)	2.9 (2.5–3.5)	0.30 (0.27–0.34)	10 (7.8–13)
Self-defined	3	0.73 (0.72–0.74)	0.71 (0.71–0.71)	4.1 (2.1–8.1)	0.32 (0.22–0.45)	13 (4.7–37)
AKIN	2	0.60 (0.55–0.61)	0.88 (0.87–0.89)	4.6 (4.1–5.1)	0.34 (0.15–0.80)	13 (5.8–29)

*ANN* artificial neural network, *GBM* gradient boosting machine, *RF* random forest, *KDIGO* Kidney Disease: Improving Global Outcomes, *AKIN* Acute Kidney Injury Network

### Sensitivity analysis

**Fig. 7:**
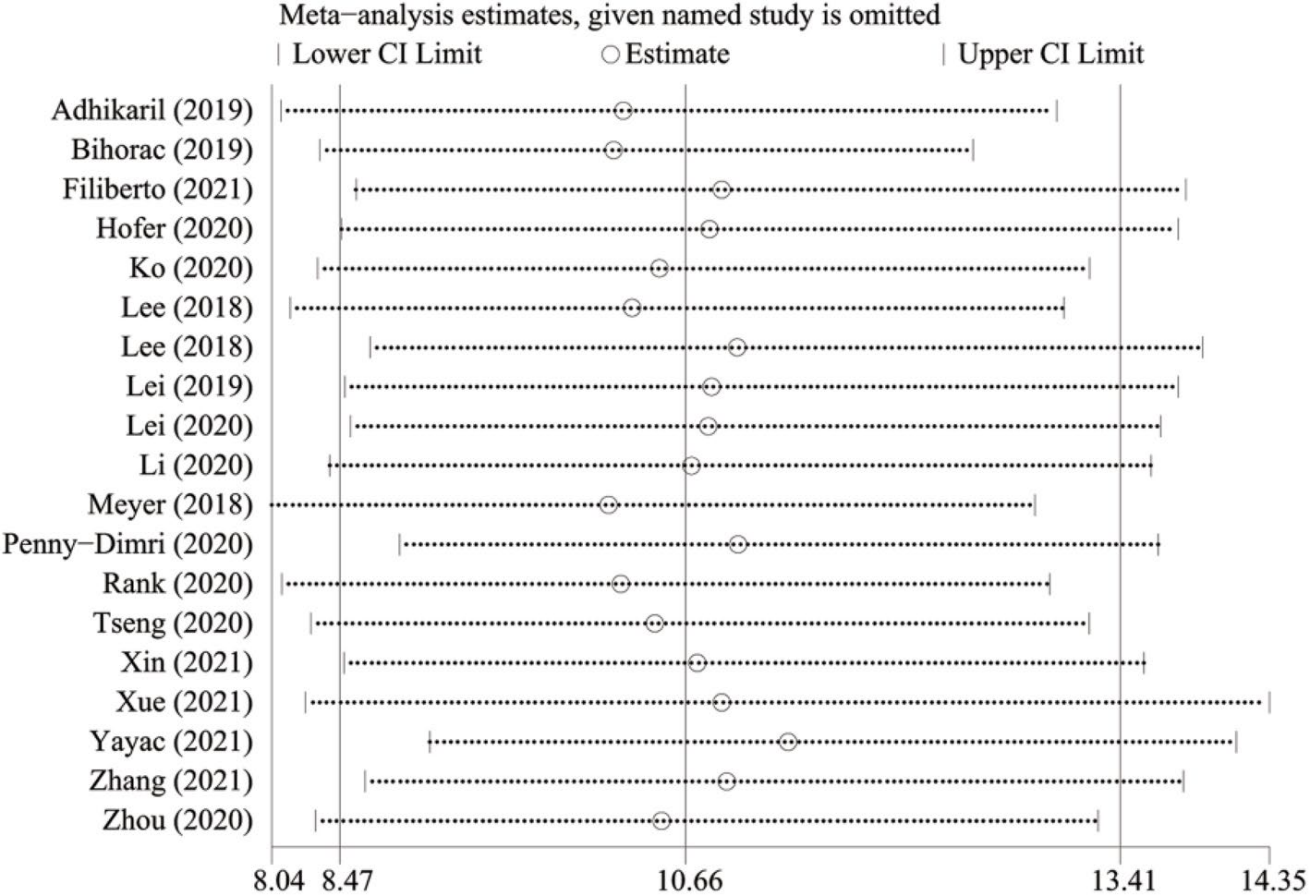
Sensitivity analysis for the prediction of AKI during the perioperative period

After excluding one study at a time, the results (Fig. 7) showed that every result is 95% within the confidence interval, combined DOR was 10.66 (95% CI: 8.47 to 13.40), which meant the outcomes of meta-analysis was robust.

### Publication Bias

**Fig. 8:**
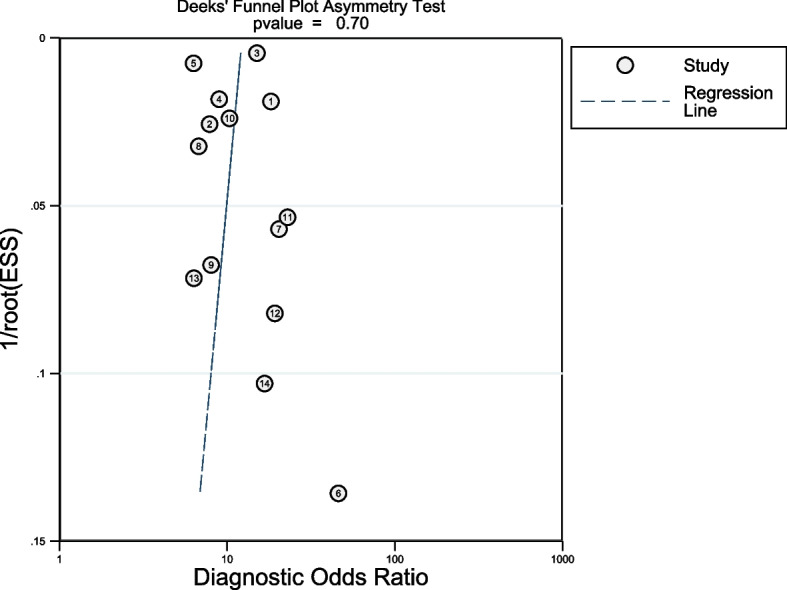
Deek’s funnel plot for the prediction of AKI during the perioperative period

Publication bias were assessing using Deek’s funnel plot for the prediction of AKI during the perioperative period (Fig. 8). The plot was grossly symmetrical with respect to the regression line. The Deek’s funnel plot asymmetry test showed no evidence of publication bias (*P* = 0.62).

## Discussion

Here, we assessed the predictive utility of artificial intelligences (AIs) in AKI during the perioperative period. Due to heterogeneous thresholds, the current optimal way to merge data is using the hierarchical summary receiver operating characteristics (HSROC) model [15]. Our study showed that the AIs can correctly detect 77% (95% CI: 0.73 to 0.81) of the patients with perioperative AKI and exclude 75% (95% CI: 0.71 to 0.80) of patients without perioperative AKI. These results presented better performance compared to the clinical scoring tools physicians used [19, 29, 35] and implied application prospects of artificial intelligences in perioperative AKI. The utlity of AKI is not only used for the prediction of AKI, but can also be used for predicting the response of AKI to specific therapies. The transition from risk stratification to therapeutic intervention is a milestone for clinical practice.

In a lot of cases, perioperative AKI are managed by non-nephrologists who may have reduced awareness of AKI and have a paucity of effective interventions [37]. In the developed countries, 30 ~ 45% of patients experienced drug-related adverse events in the non-nephrology departments [38, 39]. The delayed recognition of nephrotoxins in other departments was associated with higher mortality compared to those in the nephrology or urology department [37]. A widespread application of AI could send electronic alerts, provide a second opinion, and offer opportunities for identifying patients at risk within a time window that enables renal referral [40, 41]. Currently, how physicians would react to the early prediction made by AIs is not clear. Therefore, a prospective study based on the application of AI in clinical practice is needed.

Another important finding of this study is the robustness of the predictive performance of the AI algorithm, irrespective of the modifiers detected during the systematic review process such asAI algorithms, the type of surgery, or the criteria used in diagnosis.

Of the included 19 studies, 4 reported gradient boosted machine showed the best performance in both liver transplantation and cardiac surgery [20–22, 24]. A recent meta-analysis performed by Song and Liu et al. also found gradient boosting exhibited superior performance at predicting AKI as compared to other ML models [42]. However, after comparing the performance of seven artificial intelligence algorithms using meta-regression, no significant difference among them were found. In subgroup analysis, RF (random forest) even was superior to GBM (gradient boosting machine) with pooled sensitivity and specificity of 0.82 and 0.74 compared with 0.77 and 0.69, respectively, indicating that other algorithms might also have great potential in clinical application with predictive accuracy as good as gradient boosted machine.

[20–22, 24]The occurrence of acute kidney injury in patients receiving cardiac and vascular surgery has been widely reported, but less information was available regarding non-cardiac surgery [43], probably due to its overall lower incidence which is approximately 1% of general surgery cases [44]. Therefore, more research is required before we draw a conclusion regarding the influence of surgery type.

Our study showed that none of pre-specified subgroups showed an impact on the predictive accuracy. It suggested that the development of artificial intelligence might have hit a plateau and it might be difficult to further optimise predictive accuracy through existing methods without technological innovation. Previous studies have also shown that although physicians’ practice effectively improved, e-alerts alone could not reduce the mortality and the rate of severe AKI [45–48]. Currently, AKI diagnosis depends on changes in serum creatinine. However, novel biomarkers such as neutrophil gelatinase-associated lipocalin (NGAL), kidney injury molecule-1 (KIM-1), Cystatin C, IGFBP7, and osteopontin, as reliable measurement tools for detecting AKI have shown promising results [49–52]. NGAL or KIM-1, reportedly directly released from kidney injury might further provide methods to promptly predict an AKI event and patient prognosis in the early phase [53]. Cystatin C, a molecule with a short half-life in the serum (2 hours), is completely filtered at the glomerulus of healthy kidneys, so it might be an ideal surrogate for glomerular filtration rate and tubular cell integrity [54, 55]. Due to insufficient data about novel biomarkers on AKI risk prediction models in current studies, the real value of novel biomarkers applied in AI could not be evaluated. Further studies using novel biomarkers as input variables are essential.

The utlity of AI in AKI is not only used for the prediction of AKI, but can also be used for predicting the response of AKI to specific therapies. The transition from risk stratification to therapeutic intervention is a milestone for clinical practice [56]. Nowadays, e-alerts based on AI were widely used in conjunction with AKI care bundles to construct integrated clinical decision support system (CDS). Is the system truly rational at its current stage? Perhaps not, as the evidence base around clinical decision support system is growing but conflicting [57, 58], but if it can be tied to novel biological markers or even molecular imaging of kidney diseases, it might be.

### Strength

This reviewed included all high-quality and large-scale clinical studies published so far. Quality assessment of studies was carried out following Prediction model Risk Of Bias Assessment Tool (PROBAST) and sensitivity analysis was conducted to evaluate the robustness of our results. As a result, the artificial intelligence could prove valuable for early detection of AKI and provide aid on management decisions.

## Limitations

Despite the promising results, important limitations have to be considered. Firstly, many arguably exaggerated claims exist about AIs equivalence with (or superiority over) clinicians. It is not enough to show good predictive performance on the training set only because most show optimistic results, external validation studies are scarce, and when performed, tend to show reduced accuracy of the studied model [59]. In fact, few AI models have described any clinical effects of their use. Thus, we do not know whether it will improve (or worsen) clinical decisions [60]. Secondly, if a user strongly trusts in the e-alerts of the automatic system, they might present an indolent attitude and wait for AKI alert trigger from the model before taking action. The model requires these actions to dynamically adjust parameters and trigger the alert. This may lead to missed opportunities to mitigate or prevent AKI [61]. Thirdly, none of the 19 included studies were prospective longitudinal cohort designs, and their participant data were all from existing sources, such as existing cohort studies or routine care registries, besides, partially studies were conducted at a single centre, didn’t appropriately adjust baseline hazards or registry outcome frequency in the analysis, which had higher risk of bias and limited the reproducibility and the generalisability of the results. Fourth, AI entering the field of nephrology must adapt to legal and ethical concerns. The inability to clarify the features used because of a black-box nature conflicts with general data protection requirements [62]. Additionally, used by and serving the interests of private finance, corporations, and start-ups, AI can lead to widening social inequalities, which violates the ‘right to health legislation’ [63, 64].

## Supplementary Information

**Additional file 1.**

**Additional file 2.**

## Data Availability

All data generated or analysed during this study are included in this article.
