# Supplementary material for: Artificial intelligence for the prediction of acute kidney injury during the perioperative period: systematic review and Meta-analysis of diagnostic test accuracy
Source: BMC Nephrol. 2022 Dec 19;23:405. doi: 10.1186/s12882-022-03025-w (PMC9761969; doi:10.1186/s12882-022-03025-w)
Supplement: Supplementary file 1 — Additional file 1. [file 12882_2022_3025_MOESM1_ESM.docx]

Additional file 1

**pubmed：**

#1 “Algorithms”[Mesh] OR "Artificial Intelligence"[Mesh] OR "Decision trees"[MeSH] OR "Decision Support Systems, Clinical"[Mesh] OR "Decision Support Techniques"[Mesh] OR "Decision Support Systems, Clinical"[Mesh] OR machine learning[tiab] OR prediction algorithm*[tiab] OR prediction model*[tiab] OR neural network*[tiab] OR deep learning[tiab] OR artificial intelligence[tiab] OR AI[tiab] OR decision tree*[tiab] OR computational intelligence[tiab] OR machine intelligence[tiab] OR algorithm*[tiab] OR big data[tiab] OR bayesian[tiab] OR naïve bayes[tiab] OR k-nearest neighbour[tiab] OR decision support[tiab] OR random forest[tiab] OR support vector machine[tiab] OR SVM[tiab] OR Xgboost[tiab] OR adaboost[tiab] OR gradient boosting machine*[tiab] OR regression tree*[tiab] OR least squares[tiab] OR stepwise regression[tiab]

#2 "Acute Kidney Injury"[Mesh] OR "acute kidney injur*"[tiab] OR "Acute Renal Injur*"[tiab] OR"acute kidney damag*"[tiab] OR "acute renal damag*"[tiab] OR "acute renal insufficienc*"[tiab] OR "Acute Kidney Insufficienc*"[tiab] OR "Acute kidney failure*"[tiab] OR "Acute Renal Failure*"[tiab] OR "AKI"[tiab] OR "ARF"[tiab]

#3 "Perioperative Period"[Mesh] OR "Intraoperative Period"[Mesh] OR "Intraoperative Complications"[Mesh] OR "Postoperative Period"[Mesh] OR "Postoperative Complications"[Mesh] OR "perioperative"[tiab] OR "peri-operative"[tiab] OR "intraoperative"[tiab] OR "intra-operative"[tiab] OR "postoperative"[tiab] OR "post-operative"[tiab]

#4 Sensitivity and specificity[MeSH] OR predict*[tw] OR diagnos*[tw] OR accura*[tw]

#1 AND #2 AND #3 AND #4

182

**Embase：**

#1 'computer prediction'/exp OR 'decision tree'/exp OR 'machine learning'/exp OR 'neural network'/exp OR ‘machine learning’:ab,ti,kw OR ‘prediction algorithm*’:ab,ti,kw OR ‘prediction model*’:ab,ti,kw OR ‘neural network*’:ab,ti,kw OR ‘deep learning’:ab,ti,kw OR ‘artificial intelligence’:ab,ti,kw OR AI:ab,ti,kw OR‘decision tree*’:ab,ti,kw OR ‘computational intelligence’:ab,ti,kw OR‘machine intelligence’:ab,ti,kw OR algorithm*:ab,ti,kw OR ‘big data’:ab,ti,kw OR bayesian:ab,ti,kw OR ‘naïve bayes’:ab,ti,kw OR ‘k-nearest neighbour’:ab,ti,kw OR‘decision support’:ab,ti,kw OR ‘random forest’:ab,ti,kw OR ‘support vector machine’:ab,ti,kw OR SVM:ab,ti,kw OR Xgboost:ab,ti,kw OR adaboost:ab,ti,kw OR ‘gradient boosting machine*’:ab,ti,kw OR ‘regression tree*’:ab,ti,kw OR ‘least squares’:ab,ti,kw OR ‘stepwise regression’:ab,ti,kw

#2 'Acute Kidney failure'/exp OR 'Acute Kidney Injur*':ab,ti,kw OR 'Acute Renal Injur*':ab,ti,kw OR 'acute kidney damag*':ab,ti,kw OR 'acute renal damag*':ab,ti,kw OR 'acute renal insufficienc*':ab,ti,kw OR 'Acute Kidney Insufficienc*':ab,ti,kw OR 'Acute kidney failure*':ab,ti,kw OR 'Acute Renal Failure*':ab,ti,kw OR 'AKI':ab,ti,kw OR 'ARF':ab,ti,kw

#3 'Perioperative Period'/exp OR 'Intraoperative Period'/exp OR 'Postoperative Period'/exp OR 'Postoperative Complication'/exp OR 'perioperative':ab,ti,kw OR 'peri-operative':ab,ti,kw OR 'intraoperative':ab,ti,kw OR 'intra-operative':ab,ti,kw OR 'postoperative':ab,ti,kw OR 'post-operative':ab,ti,kw:1,828,340

#4 sensitiv* OR detect* OR accura* OR specific* OR reliab* OR positive OR negative OR diagnos*

#5 #1 AND #2 AND #3 AND #4

268

**cochrane library;**

#1 MeSH descriptor: [algorithms] explode all trees

MeSH descriptor: [artificial intelligence] explode all trees

MeSH descriptor: [dacision support systems,clinical] explode all trees

machine learning:ab,ti,kw OR prediction algorithm*:ab,ti,kw OR prediction model*:ab,ti,kw OR neural network*:ab,ti,kw OR deep learning:ab,ti,kw OR artificial intelligence:ab,ti,kw OR AI:ab,ti,kw OR decision tree*:ab,ti,kw OR computational intelligence:ab,ti,kw OR machine intelligence:ab,ti,kw OR algorithm*:ab,ti,kw OR big data:ab,ti,kw OR bayesian:ab,ti,kw OR naïve bayes:ab,ti,kw OR k-nearest neighbour:ab,ti,kw OR decision support:ab,ti,kw OR random forest:ab,ti,kw OR support vector machine:ab,ti,kw OR SVM:ab,ti,kw OR Xgboost:ab,ti,kw OR adaboost:ab,ti,kw OR gradient boosting machine*:ab,ti,kw OR regression tree*:ab,ti,kw OR least squares:ab,ti,kw OR stepwise regression:ab,ti,kw

#2 MeSH descriptor: [Acute Kidney Injury] explode all trees

Acute Kidney Injur*:ab,ti,kw OR Acute Renal Injur*:ab,ti,kw OR acute kidney damag*:ab,ti,kw OR acute renal damag*:ab,ti,kw OR acute renal insufficienc*:ab,ti,kw OR Acute Kidney Insufficienc*:ab,ti,kw OR Acute kidney failure*:ab,ti,kw OR Acute Renal Failure*:ab,ti,kw OR AKI:ab,ti,kw OR ARF:ab,ti,kw

#3 MeSH descriptor: [Perioperative Period] explode all trees

MeSH descriptor: [Intraoperative Period] explode all trees

MeSH descriptor: [Postoperative Period] explode all trees

MeSH descriptor: [Postoperative Complications] explode all trees

MeSH descriptor: [Intraoperative Complications] explode all trees

perioperative:ab,ti,kw OR peri-operative:ab,ti,kw OR intraoperative:ab,ti,kw OR intra-operative:ab,ti,kw OR postoperative:ab,ti,kw OR post-operative:ab,ti,kw

#1 and #2 and #3

review：18 trials:90

:108
